# Intensified Treatment of Tuberculous Meningitis in Adults: A Systematic Review and Meta-analysis

**DOI:** 10.1093/ofid/ofaf503

**Published:** 2025-10-07

**Authors:** Andrea Llamas-Lopez, James A Seddon, Felicia C Chow, Caryn M Upton, Sanjay K Jain, Jan-Willem Alffenaar, Daniel J Grint, Kelly Dooley, Rob Aarnoutse, Fiona V Cresswell

**Affiliations:** London School of Hygiene and Tropical Medicine, London, UK; Department of Infectious Disease, Imperial College London, London, UK; Desmond Tutu TB Centre, Department of Paediatrics and Child Health, Stellenbosch University, Stellenbosch, South Africa; Departments of Neurology and Medicine (Infectious Diseases), University of California, San Francisco, San Francisco, California, USA; University of Cape Town Lung Institute, Cape Town, Republic of South Africa; Department of Pediatrics, Johns Hopkins University, Baltimore, Maryland, USA; University of Sydney Institute for Infectious Diseases, University of Sydney, Sydney, NSW, Australia; Sydney Pharmacy School, Faculty of Medicine and Health, The University of Sydney, Camperdown, NSW, Australia; Department of Pharmacy, Westmead Hospital, Westmead, NSW, Australia; London School of Hygiene and Tropical Medicine, London, UK; Division of Infectious Diseases, Vanderbilt University Medical Center, Nashville, Tennessee, USA; Department of Pharmacy, Radboudumc, Nijmegen, The Netherlands; London School of Hygiene and Tropical Medicine, London, UK; Global Health and Infection, Brighton and Sussex Medical School, Brighton, UK; Medical Research Council/Uganda Virus Research Institute and London School of Hygiene and Tropical Medicine Uganda Research Unit, Entebbe, Uganda

**Keywords:** intensified, TBM, treatment, tuberculous meningitis

## Abstract

**Background:**

Tuberculous meningitis (TBM) remains the deadliest form of tuberculosis. Inadequate penetration of rifampicin and ethambutol into the brain and cerebrospinal fluid (CSF) may contribute to mortality. Over the last decade, research has focused on “intensified” treatment (higher-dose first-line drugs or addition of second-line drugs with good CSF penetration). This systematic review and meta-analysis evaluates the impact of intensified TBM treatment on mortality, disability, and safety.

**Methods:**

A systematic literature search was conducted of clinical trials examining intensified TBM treatments compared with a rifampicin-based standard-of-care regimen in adults. Odds ratios (ORs) were calculated using a random-effects model with mortality as the primary outcome, with OR <1 indicating lower mortality. Disability and safety were examined as secondary outcomes. Subgroup analyses included (1) higher-dose rifampicin, (2) addition of fluoroquinolones, and (3) addition of linezolid.

**Results:**

Ten trials meeting eligibility criteria, involving 1369 participants, were included. Higher-dose rifampicin (n = 1050; OR, 0.86; 95% CI, 0.54–1.35; *P* = .50), adjunctive fluoroquinolones (n = 1115; OR, 0.85; 95% CI, 0.56–1.27; *P* = .42), and linezolid (n = 79; OR, 0.73; 95% CI, 0.22–2.43; *P* = .61) did not significantly reduce TBM mortality. Due to heterogeneity in disability and safety endpoints, secondary outcomes could not be meta-analyzed.

**Conclusions:**

Current clinical trial evidence does not support the use of intensified TBM treatment in adults. However, these analyses are limited by diverse TBM case definitions, absence of MRC grading at enrollment, variable rifampicin dosing, limited data on linezolid and higher-dose isoniazid, and heterogeneous disability and safety outcomes. Use of uniform case definitions and consistent endpoints is essential to standardize data.

## Teaser

Intensified TB meningitis treatment in adults, including higher-dose rifampicin, fluoroquinolones, or linezolid, did not significantly reduce mortality. Evidence is limited by heterogeneous definitions, dosing variability, and inconsistent disability and safety outcomes, highlighting the need for standardised research methods.

An estimated 10.8 million people developed tuberculosis (TB) in 2023, with 1.25 million of these individuals dying of the disease [1]. While pulmonary TB (PTB) is the most common presentation, 17% of people with TB reported to the World Health Organization (WHO) had extrapulmonary disease [2]. Tuberculous meningitis (TBM) is one of the most challenging forms of extrapulmonary TB. A modeling study estimated that in 2019 up to 200 000 adults developed TBM, resulting in an estimated 78 000 deaths [3]. TBM is universally fatal if left untreated, and risk of death in treated TBM varies markedly by geographical setting and by HIV status. In a meta-analysis of published observational and interventional studies, overall treated TBM mortality was 24% (95% CI, 19%–29%) [4]. Mortality is considerably higher in people with HIV (53%; 95% CI, 41%–66%) than in individuals without HIV (22%; 95% CI, 4%–39%) [5]. In addition, around one-third of survivors are left with neurological disabilities [4].

Treatment of TBM has traditionally followed the same approach as that used for PTB, with the same drugs, dosages, and route of administration [6]. The treatment duration is extended to 12 months from 6 months, but this is based on very limited evidence [7]. TBM, however, presents additional therapeutic challenges due to the blood–brain and blood–cerebrospinal fluid (CSF) barriers, which lead to reduced drug exposure for certain drugs such as rifampicin and ethambutol, which have ∼5% and 20%–30% CSF penetration, respectively, when compared with plasma levels [8–12]. As a result, rifampicin, the cornerstone of TB treatment, is undetectable in the CSF in around two-thirds of TBM patients on standard-dose anti-TB treatment [11].

Given the poor outcomes seen in TBM and the limitations in drug penetration into the CSF with the currently used regimens and dosages, there has been substantial interest in optimizing drug regimens. Research to date has focused on “intensified therapy” using higher rifampicin dosing or inclusion of additional second-line drugs (such as fluoroquinolones or linezolid), which penetrate more effectively into the CSF [8, 10]. This approach has been studied in phase II and III randomized controlled trials (RCTs) in a variety of countries. However, the phase II studies have not been powered to draw conclusions about survival benefit. Clinical trials in TBM are complex and costly to conduct, so systematic evaluation and meta-analysis of studies across varied populations are important in order to provide data that may be relevant in informing treatment decisions. Such a systematic review was recently completed to evaluate optimized TBM treatment in children and provided important guidance on how to further optimize treatment in this population Therefore, this systematic review aimed to summarize and meta-analyze data from published phase II and III RCTs in adults and evaluate whether intensified treatment reduces mortality or disability from TBM.

## METHODS

### Approach and Definitions

Preferred Reporting Items for Systematic reviews and Meta-Analyses (PRISMA) guidelines were followed, and the PRISMA checklist is included as Supplementary Table 1 [13]. Intensified TBM treatment was defined as any of the following: (1) using any first-line drug at a dose that is higher than the WHO-recommended standard TB treatment (rifampicin 10 mg/kg/d, isoniazid 5 mg/kg/d, pyrazinamide 25 mg/kg/d, ethambutol 20 mg/kg/d), (2) using alternative routes of administration (eg, intravenous), (3) addition or substitution of a second-line agent [6]. Standard-of-care treatment was considered to be rifampicin-based quadruple therapy of at least 6 months’ duration and at standard dosages [6].

### Literature Search

A search was conducted of the electronic databases Ovid Medline, Ovid Embase, Ovid Global Health, Cochrane Central, and Global Index Medicus. The search was conducted on July 24, 2024. The search used terms related to TBM and antimicrobials used for TB treatment. A combination of keywords, terms, MeSh terms, and synonyms was used with adaptations to the syntax specific to each of the databases. The following search terms are examples of the ones included: “tuberculosis,” “meningeal,” “ethambutol,” “isoniazid,” “pyrazinamide,” “rifampin” or “rifampicin,” “streptomycin,” “bedaquiline,” “linezolid,” “fluoroquinolones,” “amikacin.” The complete search strategy is available in the Supplementary Data. No language restriction was applied during the search, and it was decided to restrict studies to those published after 1981, when quadruple therapy became commonplace in order to exclude RCTs conducted before the era of current therapy [14].

### Selection Criteria

To ensure the highest quality of evidence, only RCTs were included as observational studies have a high risk of confounding and bias. Eligible studies had to meet the following criteria: (i) RCT, (ii) reported clinical endpoints that included all-cause mortality in patients with TBM, (iii) adult trial population, (iv) use of intensified treatment (as defined above) in the intervention arm, and (v) use of a rifampicin-based regimen in the control arm.

Exclusion criteria included: (i) conference-only presentations without subsequent publication in a peer-reviewed journal; (ii) secondary pharmacokinetic/pharmacodynamic or drug resistance subanalysis of included studies, whereby data from the primary trial were utilized instead; (iii) studies without accessible English translation.

### Study Selection

Endnote was used as a reference manager, and all titles from the different databases were imported. Duplicate records were then identified and subsequently eliminated. The remaining publications were screened initially by title and abstract by 2 investigators independently. The remaining articles then underwent full-text review. Uncertainty or discrepancy about eligibility was resolved by discussion among the investigators.

**Figure 1.**
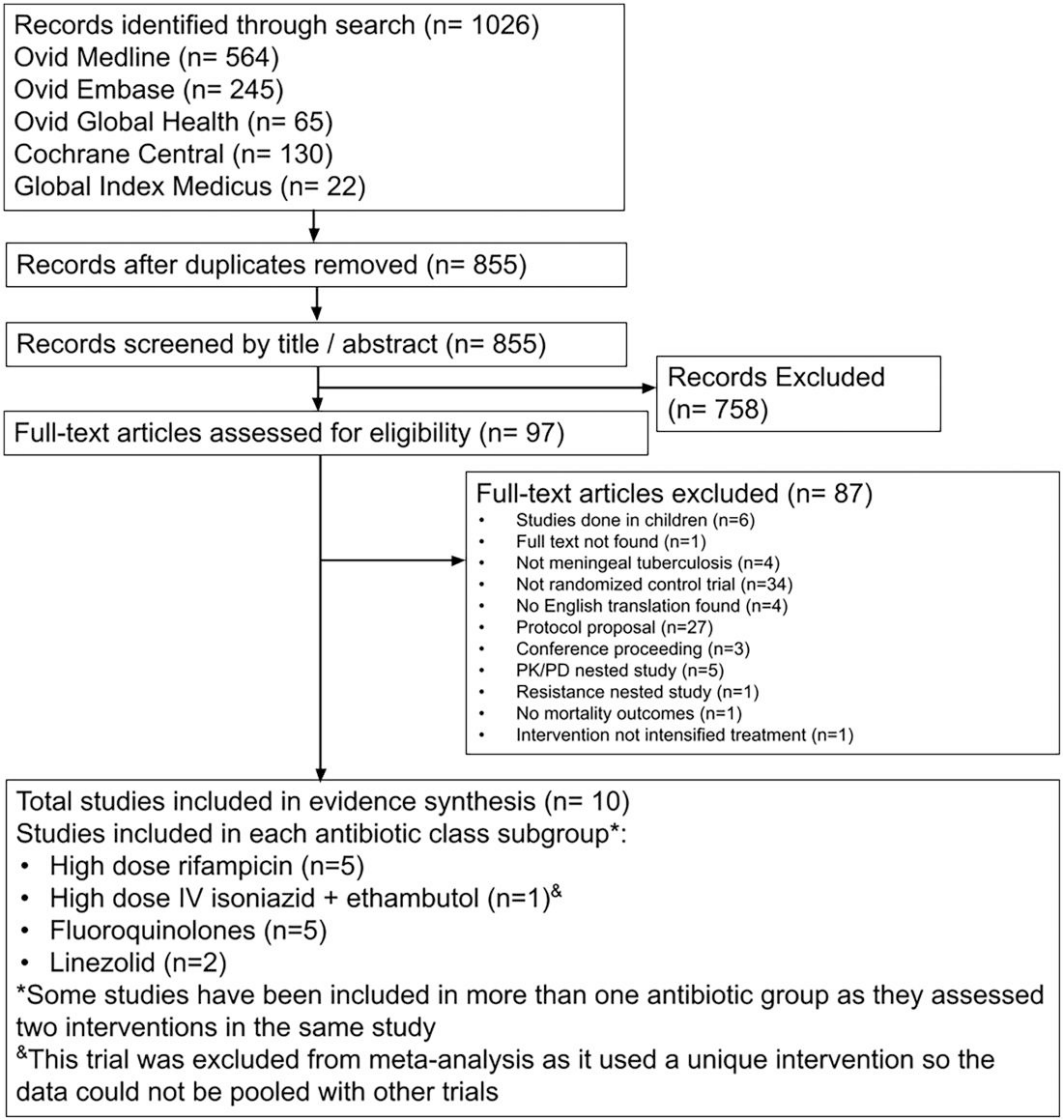
Flowchart of the study selection process. This flowchart of study selection illustrates the total number of records identified through the literature search, followed by records remaining after duplicates were removed, followed by records screened by title/abstract. The records excluded exit the flowchart with reasons for exclusion given. Full texts remaining were assessed for eligibility, and the number of full-text articles excluded is given along with reasons for exclusion. The end of the flowchart is the total number of studies included in the evidence synthesis (n = 10). Abbreviations: IV, intravenous; PK/PD, pharmacokinetic/phamacodynamic.

A PRISMA flowchart was used to summarize the selection process and highlights the number of studies included and excluded at each stage (Figure 1). According to the studies meeting eligibility criteria, 4 treatment subgroups were created by drug class in the intervention: (1) higher-dose rifampicin trials, (2) addition of fluoroquinolones, (3) addition of linezolid, and (4) higher-dose isoniazid and ethambutol trial.

### Data Extraction

Data were extracted from the eligible studies, and the information was recorded in Microsoft Excel. All fields underwent extraction by a second investigator to check for accuracy. Where any differences in data were identified, they were reviewed, and consensus was reached. Where a multi-arm trial investigated multiple drugs or doses, the data from each arm were extracted and analyzed separately. When a factorial trial design was used, all participants who received a specific intervention were included in the analysis for that intervention. Data from the intention-to-treat (ITT) analysis were extracted on the nature of the study, control and intervention regimen, primary outcome, mortality outcomes, disability (functional) outcomes, and safety (adverse events) and other outcomes.

### Risk of Bias Assessment

To assess the risk of bias, the Cochrane Collaboration's tool for assessing risk of bias, RoB-2, was used [15]. It includes 6 domains and 7 sources of bias. It has 3 categories, low, unclear, and high. A funnel plot and Harbord's test were used to assess publication bias.

### Data Analysis

Data were summarized in 2 × 2 tables, comparing mortality between the control and intervention groups. If there were 2 or more studies evaluating the same drug, data from the trials were combined in a meta-analysis. The *I*^2^ statistic was used to evaluate heterogeneity using low (<25%), moderate (25%–50%), and high (>75%) categories. A random-effects meta-analysis was used for data synthesis.

Subgroup analyses were done for the different treatment types, along with an overall pooled analysis of intensified treatment. Using STATA 18 software, binary outcomes were tabulated in order to calculate an odds ratio (OR) for the comparison between an intervention consisting of intensified TBM against a standard-of-care regimen as defined above. The results are presented in a forest plot including treatment subgroups and a pooled analysis showing ORs and 95% CIs.

## RESULTS

### Literature Search

The literature search returned 1026 records. After removing duplicates (n = 171), 855 titles and abstracts were screened, 97 full texts were reviewed for eligibility, and 10 studies were included in the final systematic review (Figure 1).

### Description of Included Studies

A total of 1369 patients were included across the 10 studies. Five studies assessed the effect of higher-dose rifampicin [11, 16–19]. Five studies evaluated the inclusion of fluoroquinolones, of which 4 examined the addition of fluoroquinolones [17, 20–22] and 1 substituted ethambutol for a fluoroquinolone [16]. Two studies assessed the addition of linezolid [19, 23], and 1 examined the optimization of isoniazid and ethambutol dosing [24]. All studies were conducted after 2009; 7 were done in Asia, 2 in Africa, and 1 in Europe (Table 1). Two of the studies [17, 18] were double-blinded, while the rest were open-label.

**Table 1.** Summary of Trials Included in the Systematic Review

Author	Year of Publication	Study Setting	No. of Participants	Study Population	HIV-Positive, No. (%)	Diagnostic Criteria Used	Study Design	Duration of Follow-up, mo	Control Regimen	Intervention	Primary Outcome	Mortality Outcomes Reported	Functional Outcomes Reported	Safety Outcomes Reported	Other Outcomes Reported
**Higher-dose rifampicin**															
Heemskerk	2016	Vietnam	817	>18 y.o. MDR-TB excluded	349 (43)	^a^	Double-blinded, placebo-controlled, 2-arm	9	WHO-recommended	In additional to standard regimen: R 5 mg/kg/d (total 15 mg/kg/d) and L 20 mg/kg/d 8-wk duration All arms followed by 7–10 RH	9-m mortality	9-mo mortality	1. Neurologic disability at 2, 6, 9 mo assessed with “simple questions” 2. “mRS” (4 tiers—good to death)	Grade 3/4 AE	Time to death or first new neurologic event (cerebellar symptoms; plegias; seizures; cranial nerve palsy; or decrease in GCS of ≥2 points for ≥2 d)
Cresswell	2021	Uganda	61	>18 y.o. MDR-TB excluded	56 (92)	^b^	Open-label, 3-arm	24 wk	WHO-recommended	In addition to standard-dose HZE: 1: IV R (20 mg/kg/d) for 14 d, then oral 35 mg/kg/d for 6 wk OR 2: Oral R 35 mg/kg/d for 8 wk All arms followed by 10 RH	R PK parameters in serum and CSF	6- and 24-wk mortality	Mean mRankin score at 8 and 24 wk	Composite safety endpoint during the first 8 wk: a) grade 3–5 AEs; or b) SAEs; or c) discontinuation of rifampicin for >5 d for any cause	Quantitative neurocognitive performance Z score at 8 and 24 wk
Dian	2018	Indonesia	60	≥14 y.o.	6 (10)	^c^	Double-blind, placebo-controlled, 3-arm	6	2HRZE/4HR	In addition to standard-dose HZE: 1: Oral R 900 mg (equiv ∼20 mg/kg/d) 2: Oral R 1350 mg (equiv ∼30 mg/kg/d) 30-d duration All arms followed by 2HRZE/4HR	R PK parameters in serum and CSF	6-mo mortality	mRS and GOS (data not presented but stated to be similar across groups)	Grade 3/4 AE	Neurological events at 3, 7, 30, 60, and 180 d
Ruslami	2013	Indonesia	60	>14 y.o. Excluded those with ATT for >7 d	7 (12)	^a^	Open-label, factorial randomizaton, 6-arm	6	2RHZE/4HR	In addition to standard-dose HZ: Randomization 1: —IV R 600 mg (equiv ∼13 mg/kg/d) or oral R 5 mg/kg/d Randomization 2: —M 400 mg or M 800 mg or E 750 mg/d 14 d duration All arms followed by 2HRZE/4HR	R PK parameters in serum and CSF Safety: grade 3/4 AE	6-mo mortality	Not done	Grade 3/4 AE	None
Davies	2023	South Africa	52	≥18 y.o. HIV-1 seropositive MDR-TB excluded	54 (100)	^a^	Open-label, 3-arm	6	WHO-recommended	In addition to standard regimen: 1: R 25 mg/kg/d (total 35 mg/kg/d) and L (1200 mg/d for 28 d, reducing to 600 mg/d for 28 d) or 2: As per 1 PLUS adjunctive aspirin 1000 mg/d Duration 56 d All arms followed by 7HR	Cumulative proportion of participants experiencing AESI or death by 56 d	8-wk and 6-mo mortality	mRS at d 56 Classified as good (0–3) vs bad (4–6)	AESI	Neurological sequelae
**Higher-dose isoniazid and ethambutol**															
Butov	2020	Ukraine	54	20–60 y.o. HIV coinfection MDR-TB excluded	54 (100)	^d^	Open-label, 2-arm	6	WHO-recommended	In addition to standard-dose RZ: IV H 500 mg/d + E 2000 mg/d PLUS RZ Duration 60 d Followed by 10HR	Effectivness of intervention	6-mo mortality	Not done	GI-related AEs	1. Sputum positivity 2. Change in CXR
**Addition of fluoroquinolones**															
Heemskerk	2016	Vietnam	817	>18 y.o. MDR-TB excluded	56 (92)	^a^	Double-blinded, placebo-controlled, 2-arm	9	WHO-recommended	In addition to standard regimen: R 5 mg/kg/d (total 15 mg/kg/d) and L 20 mg/kg/d 8-wk duration All arms followed by 7- 10RH	9-mo mortality	9-mo mortality	1. Neurologic disability at 2, 6, 9 mo assessed with “simple questions” 2. mRS (4 tiers—good to death)	Grade 3/4 AE	Time to death or first new neurologic event (cerebellar symptoms; plegias; seizures; cranial nerve palsy; or decrease in GCS of ≥2 points for ≥2 d)
Ruslami	2013	Indonesia	60	>14 y.o. Exlcuded those with treatment for TB for >7 d	7 (12)	^a^	Open-label, factorial randomizaton, 6-arm	6	2RHZE/4HR	In addition to standard-dose HZ: Randomization 1: —IV R 600 mg (equiv ∼13 mg/kg/d) or oral R 5 mg/kg/d Randomization 2: —M 400 mg or M 800 mg 14 d duration All arms followed by 2HRZE/4HR	R PK parameters in serum and CSF Safety: grade 3/4 AE	6-mo mortality	Not done	Grade 3/4 AE	None
Kalita	2016	India	57	≥15 y.o. Excluded if prior ATT for <1 mo	Not specified	^e^	Open-label, 2-arm	6	WHO-recommended	In addition to standard-dose RHZE: L 10 mg/kg/d (max 500 mg) Duration 6 mo Followed by 12HR	3- and 6- mo mortality	3- and 6-mo mortality	1. Function at 3 mo (Barthel Index score: poor = <12; partial 12–19; complete recovery ≥20) 2. Function at 6 mo	SAE/discontinuation	None
Kalita	2014	India	120	≥15 y.o. Excluded if prior ATT for <1 mo	4 (3)	^e^	Open-label	6	WHO-recommended	In addition to standard-dose RHZE: L 10 mg/kg/d (max 500 mg) with HZE Unknown duration	6-mo mortality	6-mo mortality	1. Function at 3 mo (Barthel Index score: poor = <12; partial 12–19; complete recovery ≥20) 2. Function at 6 mo	SAE/discontinuation	Change in MRI at 6 mo
Thwaites	2011	Vietnam	61	>14 y.o.	3 (5)	^f^	Open-label, 4-arm	270 d	HRZS^h^	In addition to standard-dose HRZS: 1: C 750 mg/12 h; OR 2: L 500 mg/12 h; OR 3 G 400 mg/24 h Added for the first 60 d Followed by 7HR	PK of fluoroquinolones and exposure-response relationships for the efficacy of these agents	9-mo mortality	Reported by exposure	Not reported	Plasma and CSF exposure
**Addition of linezolid**															
Davies	2023	South Africa	52	≥18 y.o. HIV-1 seropositive MDR-TB excluded	52 (100)	^a^	Open-label, 3-arm	6	WHO-recommended	In addition to standard regimen: 1: R 25 mg/kg/d (total 35 mg/kg/d) and LZD (1200 mg/d for 28 d, reducing to 600 mg/d for 28 d) or 2: As per 1 PLUS adjunctive aspirin 1000 mg/d Duration 56 d All arms followed by 7HR	Cumulative proportion of participants experiencing AESI or death by 56 d	8-wk and 6-mo mortality	mRS at d 56 Classified as good (0–3) vs bad (4–6)	AESI	Neurological sequelae
Sahib	2023	India	29	Indians ≥18 y.o. MDR-TB excluded	Not specified	^g^	Open-label, blinded outcome assessment, 2-arm	3	WHO-recommended	In addition to standard-dose RHZE: LZD (600 mg twice daily) Duration 4 wk Unknown condinuation treatment	1- and 3-mo mortality	1- and 3-mo mortality	1. mRS at 3 mo (mRS 0–2 = good outcome) 2. Median mRS	AEs/SAEs	1. Change in GCS at d 14 and d 30, (worsening/improving) 2. Radiological changes in CT scan

WHO-recommended = 2 mo RHZE/7–10 mo RH; R 10 mg/kg/d, H 5 mg/kg/d, Z 25 mg/kg/d, E 20 mg/kg/d.

Abbreviations: AE, adverse events; AESI, adverse events of significant importance; ATT, antituberculous therapy; C, ciprofloxacin; CSF, cerebrospinal fluid; CT, computed tomography; E, ethambutol; G, gatifloxacin; GCS, Glasgow Coma Scale; GOS, Glasgow Outcome Scale; H, isoniazid, L, levofloxacin; LZD, linezolid; M, moxifloxacin; MDR-TB, Multidrug resistant tuberculosis; MRI, magnetic resonance imaging scan; mRS, modified Rankin Score; PK, pharmacokinetic; R, rifampicin; S, streptomycin; SAE, serious adverse events; WHO, World Health Organization; Z, pyrazinamide.

^a^Diagnosis based on consensus criteria for suspected TBM [26].

^b^Suspected TBM (either microbiological confirmation or low CSF glucose) with TBM treatment planned.

^c^TBM diagnosis. Definite (microbiologically proven) or probable (by CSF glucose and cell count).

^d^Newly diagnosed sputum-positive DS pulmonary TB with TBM (definition of TBM not specified).

^e^Diagnosed by clinical, CSF, and MRI mixed criteria.

^f^Suspected or confirmed TBM (no definition of suspected).

^g^Suspected by the modified Ahuja criteria [27].

^h^E was substituted for S in HIV-positive adults and was added to the regimen for 3 months if previous ATT.

Inclusion criteria varied: 3 studies included participants age >14 years [16, 18], 2 included those age >15 years [20, 21], 4 included those age ≥18 years [11, 17, 19, 23], and 1 included participants who were 20–60 years old [24]. In defining TBM disease, 3 studies [16, 17, 19] used the consensus case definitions for TBM [25], 2 studies [20, 21] used the modified Ahuja criteria [26], and others employed diverse clinical, microbiological, and CSF criteria [11, 18, 22–24]. Five studies excluded participants with rifampicin-resistant TBM [11, 17, 19, 23, 24], and 3 excluded participants who had already undergone TB treatment for varying durations [16, 20, 21].

### Rationale for Meta-analysis

Mortality outcomes were consistently reported across studies. The reporting of disability outcomes and adverse events was heterogeneous between trials, meaning it was not possible to pool these data for meta-analysis (Supplementary Table 2). Therefore, only a meta-analysis using mortality data was performed.

### Risk of Bias

**Figure 2.**
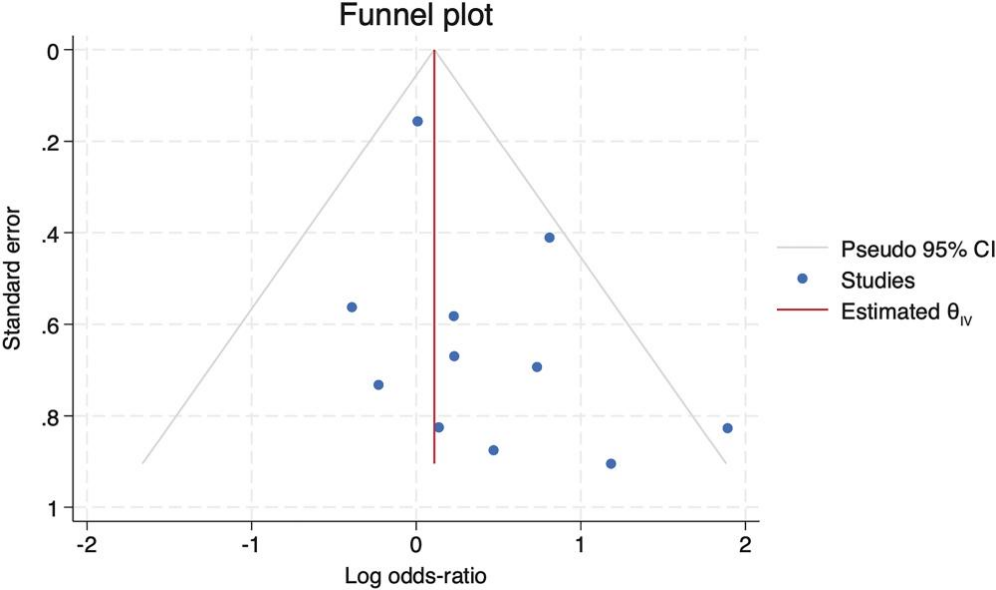
Funnel plot of studies included in the meta-analysis. This funnel plot of the studies included in the evidence synthesis shows 2 studies outside of the 95% confidence interval with a balanced number of studies within the figure.

**Figure 3.**
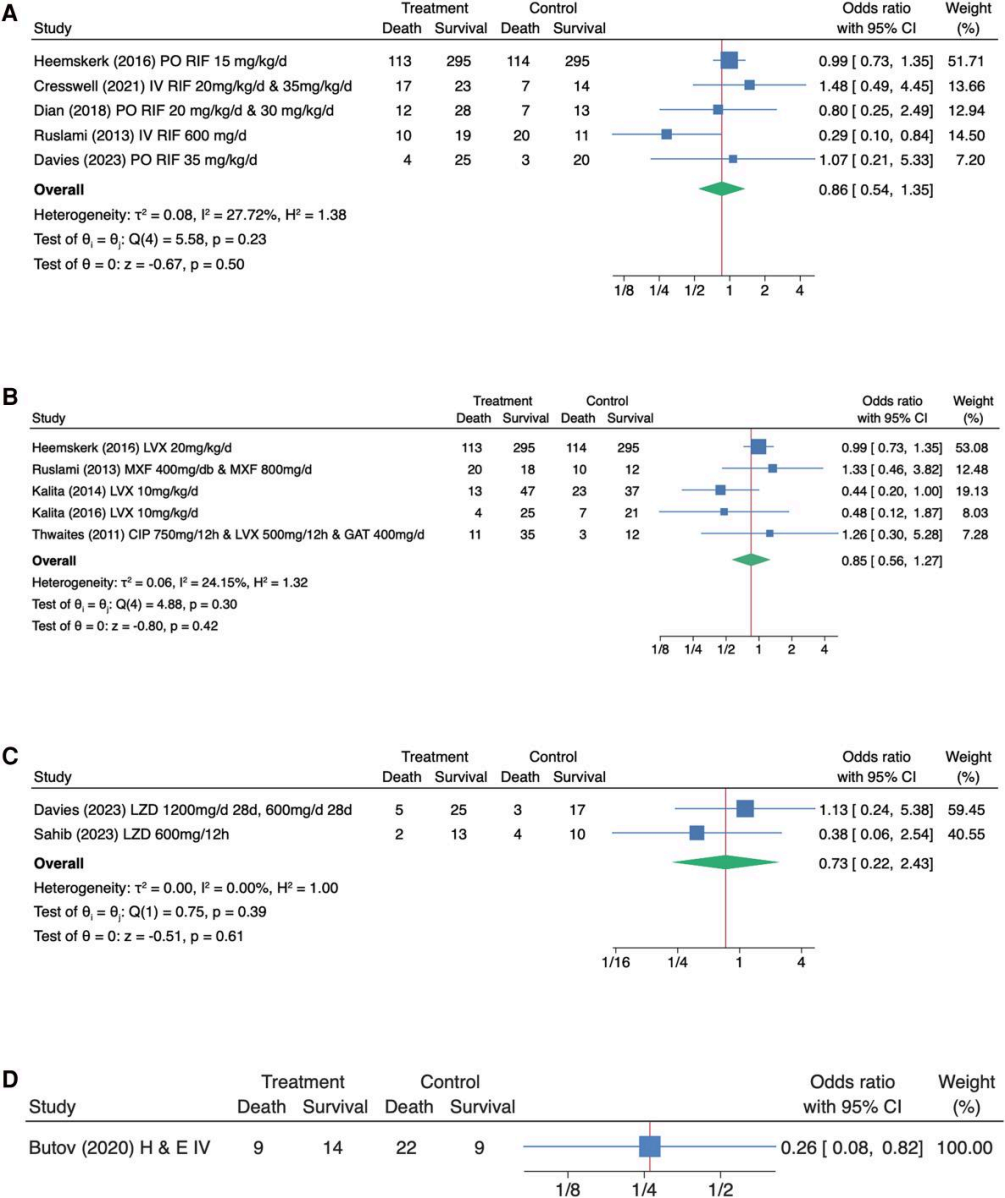
Forest plot of comparison between intervention vs control by subgroups. *A*, Use of high-dose rifampicin. *B*, Addition or substitution of fluoroquinolones. *C*, Addition of linezolid. *D*, High-dose isoniazid and ethambutol. This forest plot summarizes mortality in the intervention vs control arms, overall showing no statistically significant difference in mortality in trials assessing higher-dose rifampicin, fluoroquinolones. Abbreviations: CIP, ciprofloxacin; LVX, levofloxacin; LZD, linezolid; MXF, moxifloxacin; RIF, rifampicin. , or linezolid.

Overall, there was low risk bias across studies (Figure 2; Supplementary Figure 1), and characteristics by study are shown in Table 2. A funnel plot was created for the studies used in the meta-analysis that did not show evidence of publication bias (Figure 3). Harbord's test provided no evidence of a small-study effect.

**Table 2.** Summary of Bias of Included Studies Based on Version 2 of the Cochrane Risk-of-Bias Tool (RoB 2)

Author	Random Sequence Generation	Allocation Concealment	Blinding of Participants and Personnel	Blinding of Outcome Assessment	Incomplete Outcome Data	Other Bias
Heemskerk	Low	Low	Low	Low	Low	Low
Cresswell	Low	High	High	High	Low	Low
Dian	Low	Low	Low	Low	Unclear	Unclear
Ruslami	Low	High	High	High	Low	Unclear
Davies	Low	High	High	High	Low	Low
Butov	Low	Low	Low	Low	Low	Low
Kalita	Unclear	Unclear	Unclear	Unclear	Unclear	Unclear
Kalita	Low	Unclear	High	High	Unclear	Unclear
Thwaites	Low	High	High	High	Unclear	Unclear
Sahib	Low	Unclear	High	High	Unclear	Unclear

### Regimens Used in the Control Arm

Most of the trials used the WHO standard-of-care TB treatment regimen [11, 17, 19–21, 23, 24]. Two studies used a 6-month treatment regimen (consisting of 2 months of rifampicin, isoniazid, pyrazinamide, and ethambutol followed by a minimum of 4 months of rifampicin and isoniazid at WHO standard dosing) according to Indonesian guidelines at the time of the studies [16, 18]. One study used a regimen of rifampicin, isoniazid, pyrazinamide, and streptomycin; it also substituted streptomycin for ethambutol in HIV-positive adults and extended the regimen for 3 months in the event of previous TB treatment [22].

### Effect of Intensified Treatment on Survival

Although doses and durations of the interventional drug varied by study, due to the small number of trials, the data were pooled within treatment subgroups for the purposes of the meta-analysis, as shown in Table 1. For example, rifampicin doses of 15, 20, and 35 mg/kg/d were pooled in the high-dose rifampicin meta-analysis [11, 16–19], and any fluoroquinolones (eg, levofloxacin, ciprofloxacin, gatifloxacin, and moxifloxacin) were pooled in the fluoroquinolone subgroup [16, 17, 20–22]. For linezolid, dosing of 1200 mg for 28 days followed by 600 mg for 28 days [19] and dosing of 1200 mg for a month [23] were pooled together. A single study with high-dose isoniazid and high-dose ethambutol was also included [24].

Due to some of the studies having >1 intervention, it was decided based on the Axon et al. recommendations to pool the relevant similar interventions together (Figure 3) [27]. The meta-analysis in which each intervention was separated can be found in Supplementary Figure 2. *I*^2^ was 27.72% in the rifampicin group, suggesting moderate heterogeneity, and was <25% in the rest of the subgroups, suggesting low heterogeneity in the results.

In the high-dose rifampicin subgroup, the OR for mortality was 0.86 (95% CI, 0.54–1.35; *P* = .50). The fluoroquinolone subgroup had an OR of 0.85 compared with the standard regimen (95% CI, 0.56–1.27; *P* = .42). In the linezolid subgroup, the OR was 0.73 (95% CI, 0.22–2.43; *P* = .61). One study assessing isoniazid and ethambutol with a sample size of 54 had an OR of 0.26 (95% CI, 0.08–0.82; *P* = .02).

### Disability Outcomes

A meta-analysis of disability could not be performed due to heterogeneity in measures of functional outcomes. Studies used a variety of the modified Rankin Scale (mRS), the Barthel Index, and other measures. These measures were evaluated at inconsistent time points. One study by Sahib et al. found an improvement in disability outcomes in the intervention group when measured by the Glasgow Coma Scale (GCS) at day 30, with a median score of 14.5 compared with 12.8 in the control group (*P* = .04). However, no differences in disability outcomes were observed when measured using the mRS at 1 or 3 months [23]. Other studies, including those by Cresswell et al., Davies et al., Dian et al., Heemskerk et al., and Kalita et al. [11, 17–21], reported no significant differences in disability outcomes between trial arms.

### Safety Outcomes

Nine out of the 10 studies included reported safety data [11, 16–21, 23, 24], although there was considerable variation in the safety endpoints assessed across the studies. The most common safety outcome was the occurrence of grade 3 and 4 adverse events, which was evaluated in 3 studies [16–18]. One study used a composite safety endpoint during the first 8 weeks, including grade 3–5 adverse events, serious adverse events, or the discontinuation of rifampicin for >5 days for any reason [11]. Two studies specifically assessed serious adverse events and treatment discontinuation [20, 21], while 1 study measured adverse events of “significant importance” [19]. One study only reported gastrointestinal (GI) adverse events [24], while another measured both adverse events (grades 1–5) and severe adverse events [23].

When safety outcomes were analyzed, the study by Butov et al., which focused on optimizing isoniazid and ethambutol, found a reduction in gastrointestinal (GI) adverse events in the intervention group, with 17.3% of patients in the intervention group experiencing GI adverse events compared with 48.3% in the control group (*P* = .022) [24]. The Kalita et al. studies (2014 and 2016) showed an increase in the incidence of seizures in patients receiving levofloxacin [20, 21]. In the 2014 Kalita study, 25% of patients receiving linezolid experienced seizures compared with 6.6% in the control group (*P* = .006) [20], while in the 2016 study, 17.2% of patients in the levofloxacin group experienced seizures, with none occurring in the control group (*P* = .02) [21]. In contrast, several other studies found no differences in safety outcomes between the intervention and control groups. Studies by Cresswell et al., Davies et al., Dian et al., Heemskerk et al., Ruslami et al., and Sahib et al. all reported no differences in safety outcomes [11, 16–19], indicating that intensified treatment regimens do not cause more adverse events.

## DISCUSSION

This is the first comprehensive systematic review of intensified TBM treatments in adults. We systematically evaluated clinical trial data with a focus on mortality, disability, and safety outcomes. It was possible to meta-analyze survival data for intensification approaches. High-dose rifampicin and fluoroquinolones did not reduce mortality. No reduction in mortality with linezolid has been demonstrated, but there is currently insufficient evidence from a total of 79 participants, so no conclusions can be drawn. The most widely studied interventions to date are higher-dose rifampicin (5 trials including 1050 participants) and the addition/substitution of fluoroquinolones (5 trials including 1115 participants), followed by the addition of linezolid (2 trials). Due to variability in the disability and safety endpoints used across studies, pooling data for these outcomes was not feasible.

Rifampicin is a key drug for the treatment of TB, and notably, rifampicin does not sufficiently penetrate the CSF at standard dosages (10 mg/kg/d) [9, 10]. Rifampicin dosing of 35 mg/kg/d has been safely used in adults and children [28, 29]. Animal TBM models have shown that higher rifampicin dosing (30 mg/kg/d) improves exposures and bacterial killing [30, 31], as has been reported in clinical studies of pulmonary TB [32]. The phase II trials included in this review demonstrated significantly increased rifampicin exposures in CSF, achieving levels above the pharmacokinetic target (total area under the time concentration curve [AUC_0–24_] over the MIC) of 297 mg·h/L [11, 18, 33, 34]. However, despite target attainment, higher dosing did not improve survival. There are, however, a number of limitations to consider: Only 2 of the 5 included trials had mortality as their primary endpoint, 3 utilized high-dose rifampicin with other interventions (eg, quinolone, linezolid, aspirin) [11, 16, 18], and dosing varied from 13 mg/kg/d (intravenously) to 35 mg/kg/d (orally), as did high-dose treatment duration (14 days to 8 weeks). The largest TBM study to date, Heemskerk et al., examined a minimal increase in rifampicin dose (15 mg/kg/d) with concomitant levofloxacin [17], likely overshadowing the smaller phase II studies that used considerably higher doses (35 mg/kg/d). To account for this, the meta-analysis was run both with and without Heemskerk et al. data, with no notable change in results. This is the first robust meta-analysis of high-dose rifampicin TBM trial data as the previously published meta-analyses double-counted data from the primary trial and the pharmacokinetic analysis [35, 36]. Larger trials of high-dose rifampicin (35 mg/kg/d) are currently ongoing (eg, INTENSE-TBM [NCT041455258], HARVEST [ISRCTN15668391], IMAGINE-TBM [NCT05383742]) and will provide conclusive data in the coming 1–2 years.

Although the addition of fluoroquinolones did not show a significant impact on TBM mortality, a post hoc analysis of the impact of fluoroquinolones in isoniazid-resistant TBM showed a marked improvement in survival [37]. The WHO recommends addition of fluoroquinolone for isoniazid-monoresistant TB [38], though in many settings only rifampicin resistance is detected in real time as the Xpert MTB/Rif assay does not provide information about isoniazid resistance. Currently, there are no ongoing trials for fluoroquinolones in TBM. For linezolid, 2 small studies (79 participants total) showed a trend toward reduced mortality, but the small sample size limits any definitive conclusions. Notably, linezolid did not improve bactericidal activity of rifampicin-containing first-line regimens in animal models of TBM [39, 40] or in a phase II randomized trial substituting linezolid for ethambutol in a first-line PTB regimen [41]. Two ongoing large-scale trials (INTENSE-TBM [NCT04145258], IMAGINE-TBM [NCT05383742]) will provide more linezolid data in the coming years. There was only 1 intensified isoniazid and ethambutol study included. Although the results show a mortality reduction, it is important to note that it was a small single-center study (n = 54), so the risk of type 1 bias is considerable.

While intensified treatments for TBM were not found to reduce mortality, several other factors impact mortality beyond TB drugs. A delay in hospital presentation, diagnosis, and treatment initiation significantly impacts TBM survival due to the onset of irreversible intracerebral damage. Delays in treatment initiation result in more severe disease manifestations and worse outcomes, with mortality being ∼10% in MRC grade 1 (fully conscious) and >50% in MRC grade 3 (reduced consciousness or focal neurology) [42, 43]. For instance, in Kalita et al., the median symptom duration before enrollment was >10 weeks. Once significant intracerebral damage has occurred, even potent antibiotics with rapid bacterial killing may not change the course of disease. Further stratification of the impact of intensified treatment by MRC severity grade would be useful, though not all papers provided this data. Supportive management is also crucial for survival and must be optimized alongside antimicrobial treatment, as complications like cerebral ischemia, hyponatremia, seizures, hydrocephalus, and raised intracranial pressure contribute to mortality [44–46]. Management of these complications can require specialist expertise and equipment [47, 48]. Intracerebral inflammation is another major determinant of outcome, and corticosteroids have been shown to benefit HIV-negative patients [49, 50], though their effect in people with HIV is limited [51]. Steroids were used in all studies in this meta-analysis. A meta-analysis on adjunctive aspirin found no effect on overall mortality, but aspirin did reduce the risk of stroke [52]. Immune modulators, like anti–tumor necrosis factor agents, have shown encouraging results in case series of paradoxical reactions, though more data are needed in early disease [53, 54].

This review has limitations. Only RCTs were included to ensure rigorous study design, which reduced the number of included participants. Most studies were open-label, introducing potential bias in outcome assessment. Not all studies used the uniform case definition, and non-TB meningitis cases may have been included in trials, although this reflects a real-world challenge in TBM. Likewise, not all studies report baseline MRC grade, prohibiting comparison of outcomes by disease severity. Differences in rifampicin dosing, fluoroquinolone types, and intervention duration also added heterogeneity, but pooling across studies was justified given the small number of studies and participants. Mortality typically occurs in the first 4 weeks of treatment, so variable follow-up duration across studies is unlikely to significantly affect the mortality outcome [54]. It is also important to highlight that few studies and low power prevent further exploration of potential survival benefit seen for individual types of quinolone and for linezolid.

## CONCLUSIONS

It is important to highlight the lack of standardized outcome measures used across trials and the need for a more unified approach in future research, including consistent use of a uniform case definition, MRC grading, dosing, duration, and outcome measures. An adaptive platform trial design would more efficiently generate data to improve TBM outcomes, enabling multiple questions to be addressed in a single study. An individual patient data meta-analysis could assess the impact of specific doses or in early-stage disease, but subgroup numbers may be small. In conclusion, current RCT data show that intensified treatment does not reduce mortality in adult TBM. However, ongoing large trials of higher-dose rifampicin and linezolid may provide further insights that could influence future treatment.

## Supplementary Material

ofaf503_Supplementary_Data

## Supplementary Data

Supplementary materials are available at *Open Forum Infectious Diseases* online. Consisting of data provided by the authors to benefit the reader, the posted materials are not copyedited and are the sole responsibility of the authors, so questions or comments should be addressed to the corresponding author.
