# Supplementary material for: Intensified Treatment of Tuberculous Meningitis in Adults: A Systematic Review and Meta-analysis
Source: Open Forum Infect Dis. 2025 Oct 7;12(10):ofaf503. doi: 10.1093/ofid/ofaf503 (PMC12502660; doi:10.1093/ofid/ofaf503)
Supplement: ofaf503_Supplementary_Data [file ofaf503_supplementary_data.docx]

| **Section and Topic** | **Item #** | **Checklist item** | **Location where item is reported** |
| --- | --- | --- | --- |
| **TITLE** | | |  |
| Title | 1 | Identify the report as a systematic review. | Title |
| **ABSTRACT** | | |  |
| Abstract | 2 | See the PRISMA 2020 for Abstracts checklist. |  |
| **INTRODUCTION** | | |  |
| Rationale | 3 | Describe the rationale for the review in the context of existing knowledge. | Intro |
| Objectives | 4 | Provide an explicit statement of the objective(s) or question(s) the review addresses. | Intro |
| **METHODS** | | |  |
| Eligibility criteria | 5 | Specify the inclusion and exclusion criteria for the review and how studies were grouped for the syntheses. | Selection criteria |
| Information sources | 6 | Specify all databases, registers, websites, organisations, reference lists and other sources searched or consulted to identify studies. Specify the date when each source was last searched or consulted. | Literature search |
| Search strategy | 7 | Present the full search strategies for all databases, registers and websites, including any filters and limits used. | Appendix |
| Selection process | 8 | Specify the methods used to decide whether a study met the inclusion criteria of the review, including how many reviewers screened each record and each report retrieved, whether they worked independently, and if applicable, details of automation tools used in the process. | Study selection |
| Data collection process | 9 | Specify the methods used to collect data from reports, including how many reviewers collected data from each report, whether they worked independently, any processes for obtaining or confirming data from study investigators, and if applicable, details of automation tools used in the process. | Data extraction |
| Data items | 10a | List and define all outcomes for which data were sought. Specify whether all results that were compatible with each outcome domain in each study were sought (e.g. for all measures, time points, analyses), and if not, the methods used to decide which results to collect. | Data extraction |
|  | 10b | List and define all other variables for which data were sought (e.g. participant and intervention characteristics, funding sources). Describe any assumptions made about any missing or unclear information. | Data extraction |
| Study risk of bias assessment | 11 | Specify the methods used to assess risk of bias in the included studies, including details of the tool(s) used, how many reviewers assessed each study and whether they worked independently, and if applicable, details of automation tools used in the process. | Risk of Bias assessment |
| Effect measures | 12 | Specify for each outcome the effect measure(s) (e.g. risk ratio, mean difference) used in the synthesis or presentation of results. | Data analysis |
| Synthesis methods | 13a | Describe the processes used to decide which studies were eligible for each synthesis (e.g. tabulating the study intervention characteristics and comparing against the planned groups for each synthesis (item #5)). | Data analysis |
|  | 13b | Describe any methods required to prepare the data for presentation or synthesis, such as handling of missing summary statistics, or data conversions. | Data analysis |
|  | 13c | Describe any methods used to tabulate or visually display results of individual studies and syntheses. | Data analysis |
|  | 13d | Describe any methods used to synthesize results and provide a rationale for the choice(s). If meta-analysis was performed, describe the model(s), method(s) to identify the presence and extent of statistical heterogeneity, and software package(s) used. | Data analysis |
|  | 13e | Describe any methods used to explore possible causes of heterogeneity among study results (e.g. subgroup analysis, meta-regression). | Data analysis |
|  | 13f | Describe any sensitivity analyses conducted to assess robustness of the synthesized results. | Data analysis |
| Reporting bias assessment | 14 | Describe any methods used to assess risk of bias due to missing results in a synthesis (arising from reporting biases). | Data analysis |
| Certainty assessment | 15 | Describe any methods used to assess certainty (or confidence) in the body of evidence for an outcome. | Data analysis |
| **RESULTS** | | |  |
| Study selection | 16a | Describe the results of the search and selection process, from the number of records identified in the search to the number of studies included in the review, ideally using a flow diagram. | Literature search |
|  | 16b | Cite studies that might appear to meet the inclusion criteria, but which were excluded, and explain why they were excluded. | Literature search |
| Study characteristics | 17 | Cite each included study and present its characteristics. | Description of included studies |
| Risk of bias in studies | 18 | Present assessments of risk of bias for each included study. | Risk of Bias |
| Results of individual studies | 19 | For all outcomes, present, for each study: (a) summary statistics for each group (where appropriate) and (b) an effect estimate and its precision (e.g. confidence/credible interval), ideally using structured tables or plots. | Table 1, figure 4 |
| Results of syntheses | 20a | For each synthesis, briefly summarise the characteristics and risk of bias among contributing studies. | Table 2 Figure 2 |
|  | 20b | Present results of all statistical syntheses conducted. If meta-analysis was done, present for each the summary estimate and its precision (e.g. confidence/credible interval) and measures of statistical heterogeneity. If comparing groups, describe the direction of the effect. | Figure 4 |
|  | 20c | Present results of all investigations of possible causes of heterogeneity among study results. | Effect of intensified treatment on survival |
|  | 20d | Present results of all sensitivity analyses conducted to assess the robustness of the synthesized results. | Effect of intensified treatment on survival |
| Reporting biases | 21 | Present assessments of risk of bias due to missing results (arising from reporting biases) for each synthesis assessed. | Effect of intensified treatment on survival |
| Certainty of evidence | 22 | Present assessments of certainty (or confidence) in the body of evidence for each outcome assessed. | Effect of intensified treatment on survival |
| **DISCUSSION** | | |  |
| Discussion | 23a | Provide a general interpretation of the results in the context of other evidence. | Discussion |
|  | 23b | Discuss any limitations of the evidence included in the review. | Discussion |
|  | 23c | Discuss any limitations of the review processes used. | Discussion |
|  | 23d | Discuss implications of the results for practice, policy, and future research. | Discussion |
| **OTHER INFORMATION** | | |  |
| Registration and protocol | 24a | Provide registration information for the review, including register name and registration number, or state that the review was not registered. | N.A |
|  | 24b | Indicate where the review protocol can be accessed, or state that a protocol was not prepared. | _ |
|  | 24c | Describe and explain any amendments to information provided at registration or in the protocol. | _ |
| Support | 25 | Describe sources of financial or non-financial support for the review, and the role of the funders or sponsors in the review. | _ |
| Competing interests | 26 | Declare any competing interests of review authors. | _ |
| Availability of data, code and other materials | 27 | Report which of the following are publicly available and where they can be found: template data collection forms; data extracted from included studies; data used for all analyses; analytic code; any other materials used in the review. | _ |

**Supplementary Table 1. PRISMA checklist**

**Search Strategy**

All databases were searched in July 2024. It included studies done after 1981. No language restrictions were applied.

Databases searched and number of records retrieved

- - Ovid Medline: 564
  - Ovid Embase: 245
  - Ovid Global Health: 65
  - Cochrane Central: 130
  - Global Index Medicus: 22

Total results before duplicates removed: 1026 Results after duplicates removed: 855 **Search strategies**

**Ovid MEDLINE(R) ALL <1946 to July 24, 2024>**

| 1 | Tuberculosis, Meningeal/ | 7,545 |
| --- | --- | --- |
| 2 | ((Tubercul* or TB) adj4 (meningitis or meningeal or meninges or meningitic or pachymening* or meningoencepha*)).tw,kf. | 7,624 |
| 3 | Tuberculosis, Central Nervous System/ | 474 |
| 4 | tuberculoma, intracranial/ | 690 |
| 5 | tuberculoma/ and (intracranial* or cranial* or brain or midbrain or spinal cord* or cereb* or cns or central nervous or nervous system or pituitary or radiculomyelitis  or arachnoiditis or myeloradiculopathy or neuro* or nerve*).tw,kf. | 775 |
| 6 | ((intracranial* or cranial* or brain or midbrain or spinal cord* or cereb* or cns or  central nervous or nervous system or pituitary or radiculomyelitis or arachnoiditis or myeloradiculopathy or neuro* or nerve*) adj6 (tubercul* or TB)).tw,kf. | 5,139 |
| 7 | (Arachnoiditis/ or Meningoencephalitis/) and (tubercul* or TB).mp. | 517 |
| 8 | TBM.tw,kf. | 2,091 |
| 9 | Neurotuberculosis.tw,kf. | 139 |
| 10 | or/1-9 | 13,682 |
| 11 | (HRZE* or HRZS* or RHZE*).tw,kf. | 96 |
| 12 | (4-FDC* or 4FDC* or 4DC* or four drug*).tw,kf. | 4,089 |
| 13 | ethambutol/ or isoniazid/ or pyrazinamide/ or rifampin/ or exp Streptomycin/ | 56,548 |
| 14 | (ethambutol or myambutol or isoniazid or Isonicotinic acid or pyrazinamide or pyrazinoic acid or rifampicin or rifampin or streptomycin).tw,kf. | 62,798 |
| 15 | Bedaquiline.tw,kf. | 1,057 |
| 16 | Linezolid/ | 3,815 |
| 17 | (Linezolid or LZD or Zyvox).tw,kf. | 6,641 |
| 18 | exp Fluoroquinolones/ | 38,755 |
| 19 | (Fluoroquinolone* or Ciprofloxacin or Fleroxacin or Enoxacin or Enrofloxacin or Gatifloxacin or Gemifloxacin or Moxifloxacin or Norfloxacin or Ofloxacin or  Levofloxacin or Pefloxacin).tw,kf. | 58,173 |
| 20 | Clofazimine/ | 1,389 |

| 21 | (Clofazimine or Lamprene).tw,kf. | 1,572 |
| --- | --- | --- |
| 22 | Cycloserine/ | 2,552 |
| 23 | (Cycloserine or Seromycin or oxamycin).tw,kf. | 2,809 |
| 24 | terizidone.tw,kf. | 62 |
| 25 | (Delamanid or Deltyba).tw,kf. | 394 |
| 26 | exp Imipenem/ | 4,672 |
| 27 | (Imipenem or Imipemide).tw,kf. | 11,101 |
| 28 | Meropenem/ | 3,896 |
| 29 | (meropenem or Merrem).tw,kf. | 8,295 |
| 30 | Amikacin/ | 4,676 |
| 31 | Amikacin.tw,kf. | 10,097 |
| 32 | Prothionamide/ | 199 |
| 33 | (Prothionamide or Protionamide).tw,kf. | 256 |
| 34 | Ethionamide/ | 1,341 |
| 35 | [Ethionamide.tw](http://ethionamide.tw/),kf. | 990 |
| 36 | Aminosalicylic Acid/ | 3,932 |
| 37 | (Paser or Aminosalicylic or paraAminosalicylic).mp. or pas.ti. | 10,852 |
| 38 | Pretomanid.tw,kf. | 235 |
| 39 | or/11-38 | 174,916 |
| 40 | 10 and 39 | 2,164 |
| 41 | randomized controlled [trial.pt](http://trial.pt/). | 614,959 |
| 42 | controlled clinical [trial.pt](http://trial.pt/). | 95,536 |
| 43 | randomized.ab. | 560,081 |
| 44 | placebo.ab. | 226,090 |
| 45 | drug therapy.fs. | 2,705,718 |
|  | randomly.ab. | 363,988 |
| 47 | trial.ab. | 605,603 |
| 48 | groups.ab. | 2,264,372 |
| 49 | 41 or 42 or 43 or 44 or 45 or 46 or 47 or 48 | 5,421,619 |
| 50 | 40 and 49 [limited to RCTS using the Cochrane Highly Sensitive Search Strategy for identifying randomized trials in MEDLINE_ sensitivity-maximizing version _2023  revision] | 723 |
| 51 | limit 50 to yr="1981 -Current" | 564 |

**Embase <1981 to 2024 Week 25>**

| 1 | tuberculous meningitis/ | 6,613 |
| --- | --- | --- |
| 2 | ((Tubercul* or TB) adj4 (meningitis or meningeal or meninges or meningitic or pachymening* or meningoencepha*)).tw,kf. | 6,480 |
| 3 | central nervous system tuberculosis/ | 1,263 |
| 4 | intracranial tuberculoma/ | 887 |
| 5 | tuberculoma/ and (intracranial* or cranial* or brain or midbrain or spinal cord* or cereb* or cns or central nervous or nervous system or pituitary or radiculomyelitis or arachnoiditis or myeloradiculopathy or neuro* or nerve*).mp. | 1,925 |
| 6 | ((intracranial* or cranial* or brain or midbrain or spinal cord* or cereb* or cns or central nervous or nervous system or pituitary or radiculomyelitis or arachnoiditis or myeloradiculopathy or neuro* or nerve*) adj6 (tubercul* or TB)).tw,kf. | 6,851 |
| 7 | (arachnoiditis/ or meningoencephalitis/) and (tubercul* or TB).mp. | 1,131 |
| 8 | Neurotuberculosis.tw,kf. | 238 |
| 9 | TBM.tw,kf. | 3,493 |
| 10 | or/1-9 | 16,358 |
| 11 | (HRZE* or HRZS* or RHZE*).tw,kf. | 254 |
| 12 | (4-FDC* or 4FDC* or 4DC* or four drug*).tw,kf. | 7,519 |
| 13 | ethambutol/ or ethambutol plus isoniazid/ or ethambutol plus isoniazid plus pyrazinamide plus rifampicin/ or isoniazid/ or isoniazid plus pyrazinamide plus rifampicin/ or isoniazid plus rifampicin/ or pyrazinamide/ or rifampicin/ or rifampicin derivative/ or rifampicin plus trimethoprim/ or streptomycin/ | 159,172 |
| 14 | (ethambutol or myambutol or isoniazid or Isonicotinic acid or pyrazinamide or pyrazinoic acid or rifampicin or rifampin or streptomycin).tw,kf,dy. | 169,573 |
| 15 | bedaquiline/ | 3,226 |
| 16 | Bedaquiline.tw,kf,dy. | 3,389 |
| 17 | linezolid/ | 30,186 |
| 18 | (Linezolid or LZD or Zyvox).tw,kf,dy. | 31,225 |
| 19 | exp quinolone derivative/ | 225,587 |
| 20 | (Fluoroquinolone* or Ciprofloxacin or Fleroxacin or Enoxacin or Enrofloxacin or Gatifloxacin or Gemifloxacin or Moxifloxacin or Norfloxacin or Ofloxacin or Levofloxacin or Pefloxacin).tw,kf,dy. | 222,825 |
| 21 | clofazimine/ or clofazimine derivative/ | 8,061 |
| 22 | (Clofazimine or Lamprene).tw,kf,dy. | 8,257 |
| 23 | cycloserine/ | 7,162 |
| 24 | (Cycloserine or Seromycin or oxamycin).tw,kf,dy. | 7,561 |

| 25 | terizidone/ | 574 |
| --- | --- | --- |
| 26 | terizidone.tw,kf,dy. | 580 |
| 27 | delamanid/ | 1,382 |
| 28 | (Delamanid or Deltyba).tw,kf,dy. | 1,481 |
| 29 | imipenem/ or cilastatin plus imipenem/ or cilastatin sodium plus imipenem plus relebactam/ | 54,694 |
| 30 | (Imipenem or Imipemide).tw,kf,dy. | 56,726 |
| 31 | meropenem plus vaborbactam/ or meropenem/ | 53,629 |
| 32 | (meropenem or Merrem).tw,kf,dy. | 54,973 |
| 33 | amikacin/ | 59,909 |
| 34 | Amikacin.tw,kf,dy. | 61,731 |
| 35 | protionamide/ | 2,051 |
| 36 | (Prothionamide or Protionamide).tw,kf,dy. | 2,101 |
| 37 | ethionamide/ | 5,263 |
| 38 | Ethionamide.tw,kf,dy. | 5,395 |
| 39 | aminosalicylic acid/ | 7,788 |
| 40 | (Paser or Aminosalicylic or paraAminosalicylic).tw,kf,dy. or pas.ti. | 13,343 |
| 41 | pretomanid/ | 866 |
| 42 | Pretomanid.tw,kf,dy. | 913 |
| 43 | or/11-42 | 493,407 |
| 44 | 10 and 43 | 4,026 |
| 45 | limit 44 to conference abstracts | 360 |
| 46 | 44 not 45 | 3,666 |
| 47 | exp randomized controlled trial/ | 823,926 |
| 48 | Controlled clinical trial/ | 473,306 |
| 49 | random$.ti,ab. | 2,061,892 |

| 50 | randomization/ | 99,186 |
| --- | --- | --- |
| 51 | intermethod comparison/ | 306,858 |
| 52 | placebo.ti,ab. | 372,249 |
| 53 | (compare or compared or comparison).ti. | 605,729 |
| 54 | ((evaluated or evaluate or evaluating or assessed or assess) and (compare or compared or comparing or comparison)).ab. | 2,925,688 |
| 55 | (open adj label).ti,ab. | 115,974 |
| 56 | ((double or single or doubly or singly) adj (blind or blinded or blindly)).ti,ab. | 275,729 |
| 57 | double blind procedure/ | 216,937 |
| 58 | parallel group$1.ti,ab. | 33,623 |
| 59 | (crossover or cross over).ti,ab. | 125,842 |
| 60 | ((assign$ or match or matched or allocation) adj5 (alternate or group$1 or intervention$1 or patient$1 or subject$1 or participant$1)).ti,ab. | 431,434 |
| 61 | (assigned or allocated).ti,ab. | 509,876 |
| 62 | (controlled adj7 (study or design or trial)).ti,ab. | 470,004 |
| 63 | (volunteer or volunteers).ti,ab. | 282,683 |
| 64 | human experiment/ | 654,529 |
| 65 | trial.ti. | 419,930 |
| 66 | or/47-65 | 6,544,964 |
| 67 | (random$ adj sampl$ adj7 ("cross section$" or questionnaire$1 or survey$ or database$1)).ti,ab. not (comparative study/ or controlled study/ or randomi?ed controlled.ti,ab. or randomly assigned.ti,ab.) | 9,912 |
| 68 | Cross-sectional study/ not (exp randomized controlled trial/ or controlled clinical study/ or controlled study/ or randomi?ed controlled.ti,ab. or control group$1.ti,ab.) | 395,836 |
| 69 | (((case adj control$) and random$) not randomi?ed controlled).ti,ab. | 22,409 |
| 70 | Systematic review.ti,ab. not (trial or study).ti. | 358,103 |
| 71 | (nonrandom$ not random$).ti,ab. | 19,280 |
| 72 | "random field$".ti,ab. | 3,049 |

| 73 | (random cluster adj3 sampl$).ti,ab. | 1,649 |
| --- | --- | --- |
| 74 | (review.ab. and review.pt.) not trial.ti. | 1,202,006 |
| 75 | "we searched".ab. and (review.ti. or review.pt.) | 53,078 |
| 76 | "update review".ab. | 143 |
| 77 | (databases adj4 searched).ab. | 68,659 |
| 78 | (rat or rats or mouse or mice or swine or porcine or murine or sheep or lambs or pigs or piglets or rabbit or rabbits or cat or cats or dog or dogs or cattle or bovine or monkey or monkeys or trout or marmoset$1).ti. and animal experiment/ | 1,200,472 |
| 79 | Animal experiment/ not (human experiment/ or human/) | 2,495,822 |
| 80 | or/67-79 | 4,445,174 |
| 81 | 66 not 80 | 5,742,834 |
| 82 | 46 and 81 [limited to Cochrane EMBASE RCT filter april 2023] | 247 |
| 83 | limit 82 to yr="1981 -Current" | 245 |

**Global Health <1981 to 2024 Week 25>**

| 1 | tuberculous meningitis.sh. | 2,313 |
| --- | --- | --- |
| 2 | ((Tubercul* or TB) adj4 (meningitis or meningeal or meninges or meningitic or pachymening* or meningoencepha*)).mp. | 3,303 |
| 3 | ((intracranial* or cranial* or brain or midbrain or spinal cord* or cereb* or cns or central nervous or nervous system or pituitary or radiculomyelitis or arachnoiditis or myeloradiculopathy or neuro* or nerve*) adj6 (tubercul* or TB)).mp. | 1,985 |
| 4 | TBM.mp. | 842 |
| 5 | Neurotuberculosis.mp. | 60 |
| 6 | or/1-5 | 4,558 |
| 7 | (HRZE* or HRZS* or RHZE*).mp. | 74 |

| 8 | (4-FDC* or 4FDC* or 4DC* or four drug*).mp. [mp=abstract, title, original title, heading words, cabicodes words] | 711 |
| --- | --- | --- |
| 9 | ethambutol/ or isoniazid/ or pyrazinamide/ or rifampicin/ or streptomycin/ | 28,453 |
| 10 | (ethambutol or myambutol or isoniazid or Isonicotinic acid or pyrazinamide or pyrazinoic acid or rifampicin or rifampin or streptomycin).mp. | 34,006 |
| 11 | bedaquiline/ | 454 |
| 12 | Bedaquiline.mp. | 668 |
| 13 | linezolid/ | 4,538 |
| 14 | (Linezolid or LZD or Zyvox).mp. [mp=abstract, title, original title, heading words, cabicodes words] | 5,681 |
| 15 | exp fluoroquinolone antibiotics/ | 37,286 |
| 16 | (Fluoroquinolone* or Ciprofloxacin or Fleroxacin or Enoxacin or Enrofloxacin or Gatifloxacin or Gemifloxacin or Moxifloxacin or Norfloxacin or Ofloxacin or Levofloxacin or Pefloxacin).mp. [mp=abstract, title, original title, heading words, cabicodes words] | 39,829 |
| 17 | clofazimine/ | 676 |
| 18 | (Clofazimine or Lamprene).mp. [mp=abstract, title, original title, heading words, cabicodes words] | 1,027 |
| 19 | cycloserine/ | 659 |
| 20 | (Cycloserine or Seromycin or oxamycin).mp. [mp=abstract, title, original title, heading words, cabicodes words] | 908 |
| 21 | terizidone.mp. [mp=abstract, title, original title, heading words, cabicodes words] | 37 |
| 22 | (Delamanid or Deltyba).mp. [mp=abstract, title, original title, heading words, cabicodes words] | 263 |
| 23 | imipenem/ | 8,833 |

| 24 | (Imipenem or Imipemide).mp. [mp=abstract, title, original title, heading words, cabicodes words] | 10,501 |
| --- | --- | --- |
| 25 | meropenem/ | 6,830 |
| 26 | (meropenem or Merrem).mp. [mp=abstract, title, original title, heading words, cabicodes words] | 8,037 |
| 27 | amikacin/ | 7,447 |
| 28 | Amikacin.mp. [mp=abstract, title, original title, heading words, cabicodes words] | 8,825 |
| 29 | (Prothionamide or Protionamide).mp. [mp=abstract, title, original title, heading words, cabicodes words] | 179 |
| 30 | ethionamide/ | 560 |
| 31 | Ethionamide.mp. | 706 |
| 32 | (Paser or Aminosalicylic or paraAminosalicylic).mp. or pas.ti. | 1,307 |
| 33 | Pretomanid.mp. | 137 |
| 34 | or/7-33 | 85,214 |
| 35 | 6 and 34 | 874 |
| 36 | (rat or rats or mouse or mice or swine or porcine or murine or sheep or lambs or pigs or piglets or rabbit or rabbits or cat or cats or dog or dogs or cattle or bovine or monkey or monkeys or trout or marmoset$1).ti. and animal experiments/ | 1,186 |
| 37 | animal experiments/ not man/ | 2,292 |
| 38 | 36 or 37 | 2,538 |
| 39 | randomized controlled trials/ | 67,384 |
| 40 | randomized.ab. | 108,906 |
| 41 | placebo.ab. | 46,944 |

| 42 | randomly.ab. | 120,242 |
| --- | --- | --- |
| 43 | trial.ab. | 116,976 |
| 44 | groups.ab. | 598,567 |
| 45 | or/39-44 | 781,205 |
| 46 | 45 not 38 [RCT search filter as replicated from JF's 10.1002/jia2.25772] | 780,744 |
| 47 | 35 and 46 | 147 |
| 48 | limit 47 to yr="1981 -Current" | 65 |

**Wiley Cochrane Central Register of Controlled Trials Issue 7 of 12, July 2024**

| ID | Search | Hits |
| --- | --- | --- |
| #1 | MeSH descriptor: [Tuberculosis, Meningeal] 8 tree(s) exploded | 114 |
| #2 | (Tubercul* or TB) NEAR/3 (meningitis or meningeal or meninges or meningitic or pachymening* or meningoencepha*) | 291 |
| #3 | MeSH descriptor: [Tuberculosis, Central Nervous System] 4 tree(s) exploded | 116 |
| #4 | MeSH descriptor: [Tuberculoma, Intracranial] 5 tree(s) exploded | 1 |
| #5 | MeSH descriptor: [Tuberculoma] explode all trees | 5 |
| #6 | (intracranial* or cranial* or brain or midbrain or spinal NEXT cord* or cereb* or cns or central NEXT nervous or nervous NEXT system or pituitary or radiculomyelitis or arachnoiditis or myeloradiculopathy or  neuro* or nerve*) | 334833 |
| #7 | #5 AND #6 | 3 |
| #8 | (intracranial* or cranial* or brain or midbrain or spinal NEXT cord* or cereb* or cns or central NEXT nervous or nervous NEXT system or pituitary or radiculomyelitis or arachnoiditis or myeloradiculopathy or  neuro* or nerve*) NEAR/5 (tubercul* or TB) | 201 |
| #9 | MeSH descriptor: [Arachnoiditis] 1 tree(s) exploded | 11 |
| #10 | MeSH descriptor: [Meningoencephalitis] this term only | 15 |
| #11 | tubercul* or TB | 14853 |
| #12 | (#9 or #10) AND #11 | 5 |
| #13 | TBM | 384 |
| #14 | Neurotuberculosis | 0 |
| #15 | #1 or #2 or #3 or #4 or #7 or #8 or #12 or #13 or #14 | 694 |
| #16 | HRZE* or HRZS* or RHZE* | 76 |
| #17 | 4 NEXT FDC* or 4FDC* or 4DC* or four NEXT drug* | 5497 |
| #18 | [mh ethambutol] or [mh isoniazid] or [mh pyrazinamide] or [mh rifampin] or [mh Streptomycin] | 2162 |

| #19 | ethambutol or myambutol or isoniazid or Isonicotinic NEXT acid or pyrazinamide or pyrazinoic NEXT acid or rifampicin or rifampin or  streptomycin | 4283 |
| --- | --- | --- |
| #20 | Bedaquiline | 132 |
| #21 | [mh Linezolid] | 323 |
| #22 | Linezolid or LZD or Zyvox | 674 |
| #23 | [mh Fluoroquinolones] | 4468 |
| #24 | Fluoroquinolone* or Ciprofloxacin or Fleroxacin or Enoxacin or Enrofloxacin or Gatifloxacin or Gemifloxacin or Moxifloxacin or Norfloxacin  or Ofloxacin or Levofloxacin or Pefloxacin | 9034 |
| #25 | [mh Clofazimine] | 125 |
| #26 | Clofazimine or Lamprene | 270 |
| #27 | [mh Cycloserine] | 301 |
| #28 | Cycloserine or Seromycin or oxamycin | 538 |
| #29 | terizidone | 5 |
| #30 | Delamanid or Deltyba | 66 |
| #31 | [mh Imipenem] | 373 |
| #32 | Imipenem or Imipemide | 811 |
| #33 | [mh Meropenem] | 366 |
| #34 | meropenem or Merrem | 897 |
| #35 | [mh Amikacin] | 440 |
| #36 | Amikacin | 1045 |
| #37 | [mh Prothionamide] | 19 |
| #38 | Prothionamide or Protionamide | 55 |
| #39 | [mh Ethionamide] | 39 |
| #40 | Ethionamide | 86 |
| #41 | [mh "Aminosalicylic Acid"] | 66 |
| #42 | Paser or Aminosalicylic or paraAminosalicylic | 703 |
| #43 | (pas):ti | 136 |
| #44 | Pretomanid | 47 |
| #45 | {OR #16-#44} | 21760 |
| #46 | #15 and #45 | 165 |

with Publication Year from 1981 to 2024, with Cochrane Library publication date from Jan 1981 to Jul 2024, in Trials: 130

**Global Index Medicus** Year 1981-2024

((mening* OR intracranial* OR cranial* OR brain* OR midbrain OR "spinal cord" OR cereb* OR cns OR "central nervous" OR "nervous system" OR pituitary OR radiculomyelitis OR arachnoiditis OR myeloradiculopathy OR neuro* OR nerve*) AND (tubercul* OR TB)) OR TBM OR Neurotuberculosis

AND

(HRZE* or HRZS* or RHZE or "four drug" or ethambutol or myambutol or isoniazid or "Isonicotinic acid" or pyrazinamide or "pyrazinoic acid" or rifampicin or rifampin or streptomycin or Bedaquiline or Linezolid or LZD or Zyvox or Fluoroquinolone* or Ciprofloxacin or Fleroxacin or Enoxacin or Enrofloxacin or Gatifloxacin or Gemifloxacin or Moxifloxacin or Norfloxacin or Ofloxacin or Levofloxacin or Pefloxacin or Clofazimine or Lamprene or Cycloserine or Seromycin or oxamycin or Terizidone or Delamanid or Deltyba

or Imipenem or Imipemide or meropenem or Merrem or Amikacin or Prothionamide or Protionamide or Ethionamide or Paser or Aminosalicylic or paraAminosalicylic or pas or Pretomanid)

AND (randomized OR placebo OR randomly OR trial)

| **Author** | **Control Group** | | | | **Intervention Group** | | | |
| --- | --- | --- | --- | --- | --- | --- | --- | --- |
|  | **Total** | **Deaths** | **Functional** | **Adverse events** | **Total** | **Deaths** | **Functional** | **Adverse events** |
| **Rifampicin** |  |  |  |  |  |  |  |  |
| Heemskerk (2016) LVX 20mg/kg/d | 409 | 114 | MRC 1 - 25/178 MRC 2 - 50/178 MRC 3 - 39/71 | Any AE 229 | 408 | 113 | MRC 1 - 21/158 MRC 2 - 52/179 MRC 3 - 40/71 | Any AE 240 |
| Cresswell (2021) IV RIF 20mg/kg/d | 21 | 7 | MRS 0.86 | Composite: 15 | 20 | 7 | MRS: 0.81 | Composite: 10 |
| Cresswell (2021) PO RIF 35mg/kg/d | 21 | 7 | MRS 0.86 | Composite: 15 | 20 | 10 | MRS: 1.14 | Composite: 11 |
| Dian (2018) PO RIF 20 mg/kg/d | 20 | 7 | MRS and GOS (data not presented but stated to be similar across groups) | Grade I-II - 17 Grade III-IV- 3 | 20 | 9 | MRS and GOS (data not presented but stated to be similar across groups) | Grade I-II - 16 Grade III-IV - 8 |
| Dian (2018) PO RIF 30 mg/kg/d | 20 | 7 | MRS and GOS (data not presented but stated to be similar across groups) | Grade I-II - 17 Grade III-IV- 3 | 20 | 3 | MRS and GOS (data not presented but stated to be similar across groups) | Grade I-II - 18 Grade III-IV - 4 |
| Ruslami (2013) PO RIF 600mg/d | 31 | 11 | Not done | Grade I-II - 4 Grade III-IV- 0 | 29 | 10 | Not done | Grade I-II - 1 Grade III-IV - 3 |
| Davies (2023) PO RIF 35 mg/kg/d | 20 | 3 | MRS 0-3 :12 MRS 4-6: 5 | Grade III- 7 Grade IV- 2 | 30 | 4 | MRS 0-3 :19 MRS 4-6: 9 | Grade III - 16 Grade IV - 8 |
| **Quinolones** |  |  |  |  |  |  |  |  |
| Heemskerk (2016) LVX 20mg/kg/d | 409 | 114 | MRC 1 - 25/178 MRC 2 - 50/178 MRC 3 - 39/71 | Any AE 229 | 408 | 113 | MRC 1 - 21/158 MRC 2 - 52/179 MRC 3 - 40/71 | Any AE 240 |
| Ruslami (2013) MXF 400mg/d | 22 | 10 | Not done | Grade I-II - 4 Grade III-IV- 0 | 19 | 8 | Not done | Grade I-II - 10 Grade III-IV - 4 |
| Ruslami (2013) MXF 800mg/d | 22 | 10 | Not done | Grade I-II - 4 Grade III-IV- 0 | 19 | 12 | Not done | Grade I-II - 7 Grade III-IV - 6 |
| Kalita (2014) LVX 10mg/kg/d | 60 | 23 | Poor: 5 Partial: 6 Complete: 22 | Seizure: 4 Myoclonus: 0 Encephalopathy: 0 Discontinuation due to SAEs: 4 | 60 | 13 | Poor: 1 Partial: 7 Complete: 25 | Seizure: 15 Myoclonus: 3 Encephalopathy: 1 Discontinuation due to SAEs: 16 |
| Kalita (2016) LVX 10mg/kg/d | 28 | 7 | Poor: 10 Good: 18 | Composite: 13 | 29 | 4 | Poor: 9 Good: 20 | Composite: 10 |
| Thwaites (2011) CIP 750mg/12h | 15 | 3 | Reported by exposure | Not reported | 16 | 3 | Reported by exposure | Not reported |
| Thwaites (2011) LVX 500mg/12h | 15 | 3 | Reported by exposure | Not reported | 15 | 6 | Reported by exposure | Not reported |
| Thwaites (2011) GAT 400mg/d | 15 | 3 | Reported by exposure | Not reported | 15 | 2 | Reported by exposure | Not reported |
| **Linezolid** |  |  |  |  |  |  |  |  |
| Davies (2023) LZD 1200mg 28d, 600mg 28d | 20 | 3 | MRS 0-3 :12 MRS 4-6: 5 | Grade III- 7 Grade IV- 2 | 30 | 5 | Grade III - 16 Grade IV - 8 | MRS 0-3 :19 MRS 4-6: 9 |
| Sahib (2023) LZD 600mg/12h | 14 | 4 | mRS0-2: 4 mRS 0-3: 7 | Visual acuity: 5 Papilledema: 2 Optic atrophy: 3 Liver disfunction: 5 Bleeding: 1 Myelosupression: 0 Tingling/numbness/pain: 1 | 15 | 2 | mRS0-2: 7 mRS 0-3: 8 | Visual acuity: 4 Papilledema: 0 Optic atrophy: 2 Liver disfunction: 4 Bleeding: 0 Myelosupression: 1 Tingling/numbness/pain: 2 |
| **Isoniazid and Ethambutol** |  |  |  |  |  |  |  |  |
| Butov | 31 | 22 | Not done | GI AEs: 15 | 23 | 9 | Not done | GI AEs: 4 |
| MRS: Mean modified rankin scores | | | | | | | | |

**Supplementary Table 2.** **Numerical outcomes relating to overall mortality, safety, and disability outcomes.**


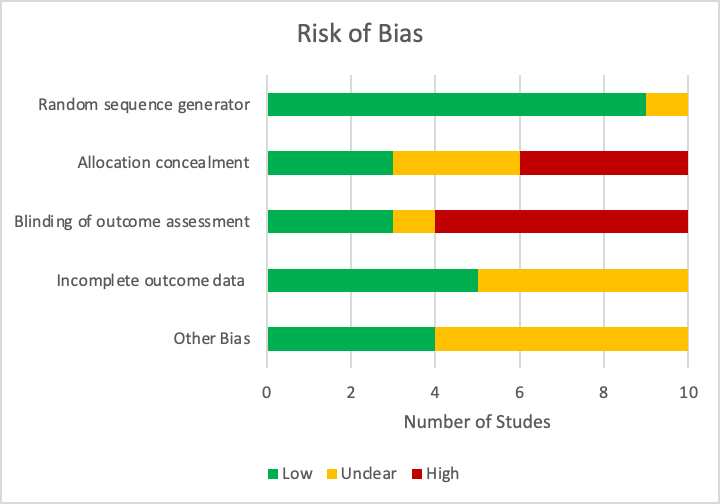


**Supplementary Figure 1: Overall risk of bias in included studies evaluating intensified treatment for tuberculous meningitis.**


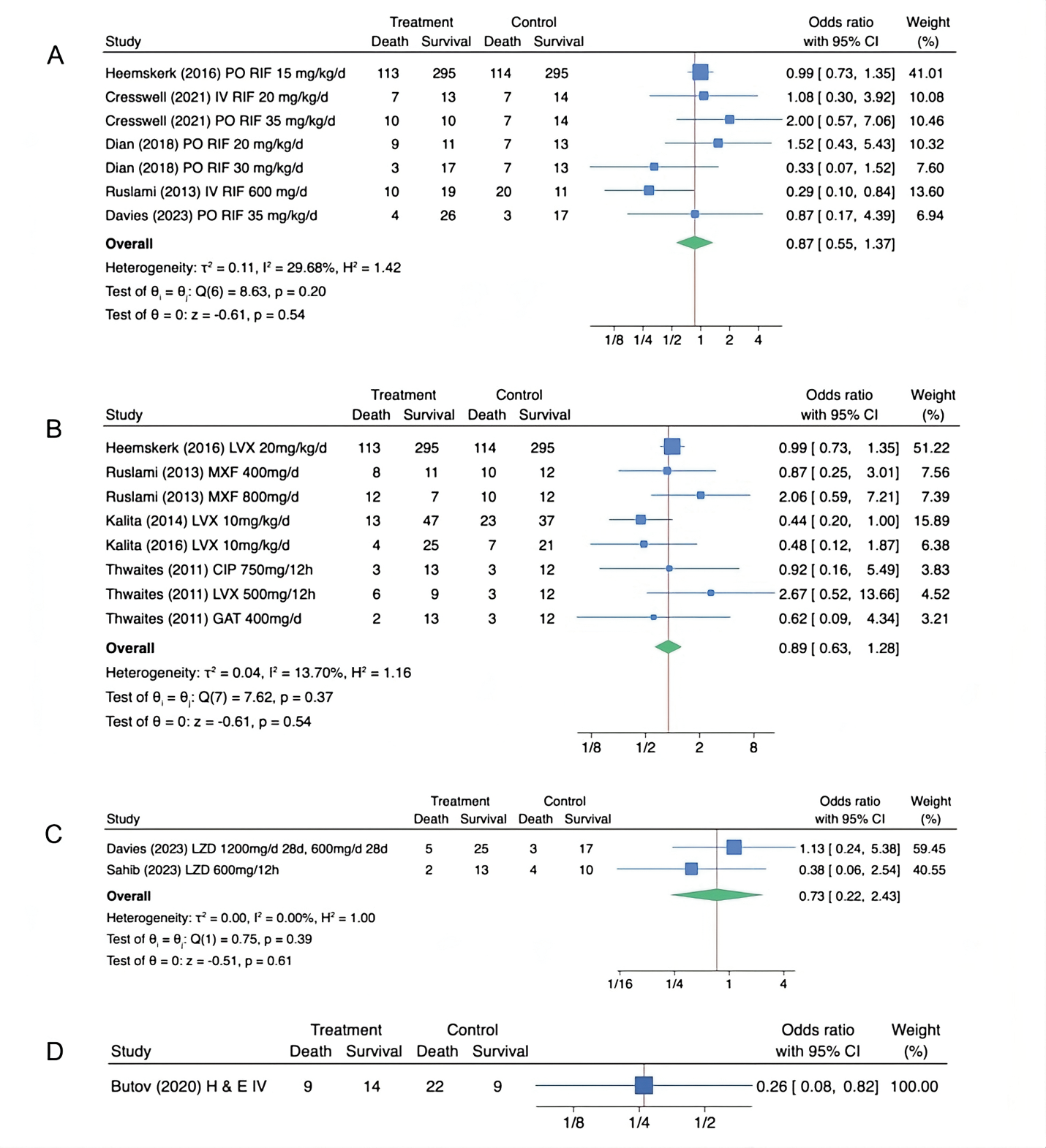


**Supplementary Figure 2: Forest plot of comparison of mortality between individual interventions separated by dose and route of administration versus controls. A. Use of high-dose rifampicin. B. Addition or substitution with fluoroquinolones. C. Addition of linezolid. D. High dose isoniazid and ethambutol.**
